# Supplementary material for: Identification of Arctic Food Fish Species for Anthropogenic Contaminant Testing Using Geography and Genetics
Source: Foods. 2020 Dec 8;9(12):1824. doi: 10.3390/foods9121824 (PMC7764770; doi:10.3390/foods9121824)
Supplement: Supplementary file 1 [file foods-09-01824-s001.pdf]

**Identification of Arctic food fish species for anthropogenic contaminant testing using geography and genetics- Supplemental Material**

Virginia K. Walker1,2 Virginia K. Walker[1,2]*, Pranab Das[1], Peiwen Li[1], Stephen C. Lougheed[1], Kristy Moniz[1], Stephan Schott[3], James Qitsualik[4], and Iris Koch[1,5]

**Supplemental Figures and Statistics**

**Correlations and Statistical Analysis**

As noted in Methods, non-parametric Spearman rank-order correlation analysis was conducted. The Spearman test examines the ranks of values and whether elements move together (coefficient, ϱ, of 1), move in opposite directions (ϱ = -1), or have no relationship (ϱ = 0). The main focus of this statistical testing was to examine PCB correlations (N = 101). The correlation analysis was repeated for Hg, As, and elements that were correlated with Hg and As in the PCB correlation analysis, using the full inorganic dataset, for samples where all the elements were available (N = 531).

The correlation plot for the PCB dataset is shown in Figure S1, with colours indicating a statistically significant correlation: red shades indicating positive correlations, blue shades indicating negative correlations, and darker colours indicating stronger correlations. Variables of interest are indicated with green shading (PCBs, Hg and As). Nutritional elements are indicated with yellow shading. Elements with less than 50% detectable values are indicated with grey shading, and elements with 0–4 detectable values were not included in the analysis.

Total PCBs correlate with age, Se, Hg and Na, and negatively with Cr and V, but no correlations are seen with % lipid. Therefore, fish that have higher PCB concentrations are older, and also have higher Se, Hg and Na concentrations, and lower Cr and V concentrations. More correlations are seen when PCBs are expressed on a % lipid basis (T-PCBlip), and these correlations include those with As and some of the nutritional elements. It should be noted that the negative correlation of T-PCBlip with % lipid is an artefact of the calculation to express PCBs on a lipid weight basis: T-PCBlip = T-PCB/fraction lipid. T-PCBlip correlations with nutritional elements (zinc (Zn), sulfur (S), Na, magnesium (Mg), phosphorus (P), potassium (K), and manganese (Mn)) are a consequence of the negative correlation of these elements with % lipid. That is, less fatty fish, which result in higher T-PCBlip values, have higher concentrations of these nutritional elements.

Hg is correlated positively with age and negatively with % lipid, meaning that older fish and less fatty fish are associated with higher Hg concentrations. Older fish are also likely to be associated with higher As concentrations and less fat. Nutritional elements (Ca, Zn, Co, S, Na, K, Mg, P, Mn) correlate well with each other; Ti, Rb and Sr, which are naturally occurring elements in the environment, correlate well with this group as well. Thallium (Tl, usually considered a contaminant), correlates well with several nutritional elements, in particular those that form positively charged ions in biological systems (*e.g.*, K, Na, Mg).

Correlations are seen between elements with 50% or fewer detectable values (but virtually none with PCBs), but any correlations seen are likely attributable to a preponderance of detection limit substitutions (i.e., non-detects), and should not be considered to be statistically robust.

When Hg and As correlations were examined with the full dataset of inorganic elements, similar results were found as for the PCB dataset. Hg was correlated with age and As, as well as the nutritional elements Ca, Mg, Mn, P, K, Na, S, and Zn, and negatively with Cr, Cu and V. Hg was interestingly not correlated either positively or negatively with Se even though Hg and Se are thought to be antagonistic [88]. Of the nutritional elements that Hg was correlated with, the only Cu and Cr (negative), and Na and S (positive) were correlated with age. Thus, the correlations of Hg with nutritional elements was not necessarily a consequence of higher nutritional element content with age. However, most of the

nutritional elements were strongly correlated with each other. Cu, Cr and V had many negative correlations with other elements; the reasons for negative correlations between nutritional elements are not known and homeostasis (and even the precise function) of nutritional elements in specific biological organisms is a continuing area of research.

Arsenic was correlated with age, Hg (as mentioned previously), Na and Se, and negatively with Ca, Cu, Cr, and V. Both Hg (all forms) and inorganic As forms interact with S in biological systems, and in some cases S-containing proteins may be produced as a toxic or adaptive response to these two elements and other metals (*e.g.*, metallothioneins, glutathione) [89, 90]; this may explain the correlation between Hg and S. However, the lack of correlation between As and S may suggest that the form of As is not biologically reactive, and this is thought to be the reason for the non-toxicity of arsenobetaine [72], which is likely the form of As in samples with increased concentrations, as noted in the Discussion.

| Var | Age | %lipid | T-PCBs | T-PCBLip | DL PCBs | Hg | As | Ni | Cu | Cr | Se | Fe | Zn | Ca | S | Na | Mg | P | K | Mn | Co | V | Ti | Rb | Sr | Tl | La | Ba | Cd | U | Te | Ce | B | Pr | Be | Nd | Pb | Pt | Li | Sa |
|---|---|---|---|---|---|---|---|---|---|---|---|---|---|---|---|---|---|---|---|---|---|---|---|---|---|---|---|---|---|---|---|---|---|---|---|---|---|---|---|---|
| Age | | -0.3 | 0.3 | 0.4 | 0.2 | 0.6 | 0.4 | -0.1 | -0.4 | -0.2 | 0.1 | 0.1 | 0.1 | 0.0 | 0.3 | 0.4 | 0.1 | 0.2 | 0.2 | 0.1 | 0.2 | 0.0 | 0.2 | 0.2 | 0.1 | -0.1 | 0.2 | 0.0 | 0.2 | 0.0 | 0.0 | 0.1 | -0.1 | 0.1 | -0.1 | 0.0 | -0.3 | -0.1 | -0.1 | 0.0 |
| %lipid | -0.3 | | 0.0 | -0.5 | -0.1 | -0.5 | -0.2 | -0.1 | 0.1 | 0.0 | -0.2 | -0.2 | -0.4 | -0.3 | -0.8 | -0.6 | -0.6 | -0.7 | -0.7 | -0.5 | -0.3 | 0.0 | -0.6 | -0.7 | -0.2 | -0.3 | -0.1 | -0.1 | 0.0 | 0.0 | 0.1 | 0.0 | 0.1 | 0.1 | 0.2 | 0.1 | 0.2 | 0.2 | 0.2 | 0.1 |
| T-PCBs | 0.3 | 0.0 | | 0.8 | 0.9 | 0.2 | 0.1 | 0.0 | -0.1 | -0.3 | 0.3 | 0.0 | 0.2 | 0.0 | 0.2 | 0.3 | -0.1 | 0.1 | 0.0 | -0.1 | 0.0 | -0.2 | 0.0 | 0.1 | -0.1 | 0.1 | -0.1 | -0.1 | -0.1 | -0.2 | -0.1 | -0.1 | 0.1 | -0.1 | -0.2 | -0.1 | -0.1 | -0.1 | -0.1 | 0.0 |
| T-PCBLip | 0.4 | -0.5 | 0.8 | | 0.8 | 0.4 | 0.2 | 0.0 | -0.1 | -0.2 | 0.3 | 0.1 | 0.3 | 0.2 | 0.5 | 0.5 | 0.3 | 0.4 | 0.4 | 0.2 | 0.2 | -0.1 | 0.3 | 0.4 | 0.0 | 0.2 | 0.0 | 0.0 | -0.1 | -0.1 | -0.1 | -0.1 | 0.0 | -0.1 | -0.3 | -0.1 | -0.1 | -0.1 | -0.2 | -0.1 |
| DL PCBs | 0.2 | -0.1 | 0.9 | 0.8 | | 0.3 | 0.1 | 0.0 | -0.1 | -0.2 | 0.2 | 0.1 | 0.2 | 0.1 | 0.3 | 0.4 | 0.1 | 0.2 | 0.1 | 0.1 | 0.0 | -0.2 | 0.1 | 0.2 | 0.0 | 0.1 | -0.1 | 0.0 | -0.1 | -0.2 | -0.1 | -0.1 | 0.0 | -0.1 | -0.3 | -0.1 | -0.1 | -0.2 | -0.2 | -0.1 |
| Hg | 0.6 | -0.5 | 0.2 | 0.4 | 0.3 | | 0.2 | -0.1 | -0.3 | -0.3 | -0.1 | -0.1 | 0.4 | 0.2 | 0.6 | 0.6 | 0.2 | 0.4 | 0.3 | 0.3 | 0.1 | -0.3 | 0.3 | 0.4 | 0.1 | 0.1 | 0.1 | 0.0 | 0.0 | -0.1 | -0.2 | 0.0 | 0.0 | 0.0 | -0.2 | -0.1 | -0.3 | -0.1 | -0.2 | -0.1 |
| As | 0.4 | -0.2 | 0.1 | 0.2 | 0.1 | 0.2 | | -0.1 | -0.1 | -0.4 | 0.4 | 0.0 | -0.1 | 0.0 | 0.1 | 0.1 | 0.0 | 0.1 | 0.1 | 0.0 | 0.2 | 0.0 | 0.1 | -0.1 | 0.3 | -0.4 | 0.3 | 0.2 | 0.0 | 0.1 | 0.1 | 0.2 | -0.1 | 0.1 | 0.0 | 0.1 | -0.1 | 0.0 | 0.0 | 0.1 |
| Ni | -0.1 | -0.1 | 0.0 | 0.0 | 0.0 | -0.1 | -0.1 | | 0.1 | 0.5 | -0.1 | 0.3 | 0.2 | 0.4 | 0.0 | 0.1 | 0.2 | 0.2 | 0.1 | 0.5 | 0.4 | 0.3 | 0.0 | 0.2 | 0.4 | 0.3 | 0.1 | 0.3 | -0.1 | -0.1 | 0.0 | -0.2 | -0.3 | -0.2 | -0.1 | -0.2 | -0.2 | -0.3 | -0.2 | -0.3 |
| Cu | -0.4 | 0.1 | -0.1 | -0.1 | -0.1 | -0.3 | -0.1 | 0.1 | | 0.2 | 0.3 | 0.4 | -0.1 | -0.1 | -0.2 | -0.2 | -0.2 | -0.2 | -0.2 | -0.1 | -0.4 | -0.1 | -0.1 | -0.2 | -0.3 | 0.0 | -0.2 | -0.2 | 0.0 | -0.3 | -0.2 | -0.2 | 0.1 | -0.2 | 0.0 | -0.3 | 0.1 | -0.1 | -0.1 | -0.2 |
| Cr | -0.2 | 0.0 | -0.3 | -0.2 | -0.2 | -0.3 | -0.4 | 0.5 | 0.2 | | -0.2 | 0.4 | -0.1 | 0.1 | 0.0 | -0.2 | 0.0 | 0.0 | 0.0 | 0.1 | 0.1 | 0.3 | -0.1 | 0.0 | -0.1 | 0.2 | -0.1 | 0.0 | -0.1 | 0.0 | 0.1 | 0.0 | 0.1 | 0.0 | 0.2 | 0.0 | 0.0 | 0.1 | 0.0 | 0.0 |
| Se | 0.1 | -0.2 | 0.3 | 0.3 | 0.2 | -0.1 | 0.4 | -0.1 | 0.3 | -0.2 | | 0.3 | 0.1 | -0.1 | 0.1 | 0.1 | 0.1 | 0.0 | 0.1 | 0.0 | 0.0 | 0.0 | 0.1 | 0.0 | -0.1 | 0.0 | 0.1 | 0.1 | 0.1 | -0.1 | 0.0 | 0.0 | -0.2 | -0.1 | -0.1 | -0.1 | 0.1 | 0.1 | -0.1 | 0.0 |
| Fe | 0.1 | -0.2 | 0.0 | 0.1 | 0.1 | -0.1 | 0.0 | 0.3 | 0.4 | 0.4 | 0.3 | | 0.3 | 0.2 | 0.1 | 0.2 | 0.1 | 0.1 | 0.1 | 0.3 | 0.3 | 0.4 | 0.3 | 0.1 | 0.1 | 0.1 | 0.3 | 0.3 | 0.3 | 0.1 | 0.0 | 0.0 | -0.3 | 0.0 | -0.1 | 0.0 | 0.1 | 0.0 | -0.2 | 0.0 |
| Zn | 0.1 | -0.4 | 0.2 | 0.3 | 0.2 | 0.4 | -0.1 | 0.2 | -0.1 | -0.1 | 0.1 | 0.3 | | 0.5 | 0.4 | 0.6 | 0.4 | 0.5 | 0.4 | 0.5 | 0.4 | 0.1 | 0.3 | 0.5 | 0.3 | 0.5 | 0.2 | 0.3 | 0.1 | -0.1 | -0.2 | -0.1 | -0.3 | -0.2 | -0.4 | -0.2 | 0.0 | -0.2 | -0.4 | -0.2 |
| Ca | 0.0 | -0.3 | 0.0 | 0.2 | 0.1 | 0.2 | 0.0 | 0.4 | -0.1 | 0.1 | -0.1 | 0.2 | 0.5 | | 0.3 | 0.4 | 0.4 | 0.5 | 0.3 | 0.8 | 0.4 | 0.3 | 0.4 | 0.4 | 0.8 | 0.4 | 0.2 | 0.5 | -0.1 | 0.1 | 0.1 | -0.1 | -0.2 | -0.1 | -0.3 | -0.1 | -0.1 | -0.1 | -0.3 | -0.2 |
| S | 0.3 | -0.8 | 0.2 | 0.5 | 0.3 | 0.6 | 0.1 | 0.0 | -0.2 | 0.0 | 0.1 | 0.1 | 0.4 | 0.3 | | 0.7 | 0.7 | 0.8 | 0.8 | 0.5 | 0.2 | -0.1 | 0.5 | 0.8 | 0.2 | 0.4 | 0.1 | 0.0 | -0.1 | 0.0 | -0.2 | 0.0 | -0.1 | -0.1 | -0.3 | -0.1 | -0.2 | -0.2 | -0.3 | -0.2 |
| Na | 0.4 | -0.6 | 0.3 | 0.5 | 0.4 | 0.6 | 0.1 | 0.1 | -0.2 | -0.2 | 0.1 | 0.2 | 0.6 | 0.4 | 0.7 | | 0.4 | 0.6 | 0.5 | 0.4 | 0.3 | -0.1 | 0.4 | 0.6 | 0.3 | 0.3 | 0.2 | 0.2 | 0.1 | -0.1 | -0.2 | 0.0 | -0.2 | -0.1 | -0.3 | -0.1 | -0.1 | -0.3 | -0.3 | -0.1 |
| Mg | 0.1 | -0.6 | -0.1 | 0.3 | 0.1 | 0.2 | 0.0 | 0.2 | -0.2 | 0.0 | 0.1 | 0.1 | 0.4 | 0.4 | 0.7 | 0.4 | | 0.8 | 0.9 | 0.6 | 0.4 | 0.2 | 0.5 | 0.8 | 0.3 | 0.5 | 0.2 | 0.3 | -0.2 | 0.1 | -0.1 | -0.1 | -0.3 | -0.1 | -0.3 | -0.1 | -0.1 | -0.3 | -0.3 | -0.2 |
| P | 0.2 | -0.7 | 0.1 | 0.4 | 0.2 | 0.4 | 0.1 | 0.2 | -0.2 | 0.0 | 0.0 | 0.1 | 0.5 | 0.5 | 0.8 | 0.6 | 0.8 | | 0.8 | 0.6 | 0.3 | 0.1 | 0.5 | 0.8 | 0.3 | 0.5 | 0.1 | 0.2 | -0.2 | 0.1 | -0.2 | -0.1 | -0.1 | -0.1 | -0.3 | -0.1 | -0.2 | -0.4 | -0.3 | -0.2 |
| K | 0.2 | -0.7 | 0.0 | 0.4 | 0.1 | 0.3 | 0.1 | 0.1 | -0.2 | 0.0 | 0.1 | 0.1 | 0.4 | 0.3 | 0.8 | 0.5 | 0.9 | 0.8 | | 0.5 | 0.3 | 0.2 | 0.5 | 0.9 | 0.3 | 0.5 | 0.2 | 0.2 | -0.1 | 0.1 | -0.1 | 0.0 | -0.2 | -0.1 | -0.2 | -0.1 | -0.1 | -0.3 | -0.3 | -0.2 |
| Mn | 0.1 | -0.5 | -0.1 | 0.2 | 0.1 | 0.3 | 0.0 | 0.5 | -0.1 | 0.1 | 0.0 | 0.3 | 0.5 | 0.8 | 0.5 | 0.4 | 0.6 | 0.6 | 0.5 | | 0.6 | 0.5 | 0.5 | 0.5 | 0.7 | 0.4 | 0.3 | 0.4 | -0.1 | 0.2 | 0.0 | -0.1 | -0.3 | -0.1 | -0.3 | -0.1 | -0.1 | -0.2 | -0.4 | -0.2 |
| Co | 0.2 | -0.3 | 0.0 | 0.2 | 0.0 | 0.1 | 0.2 | 0.4 | -0.4 | 0.1 | 0.0 | 0.3 | 0.4 | 0.4 | 0.2 | 0.3 | 0.4 | 0.3 | 0.3 | 0.6 | | 0.6 | 0.4 | 0.4 | 0.6 | 0.3 | 0.5 | 0.6 | 0.1 | 0.3 | 0.3 | 0.2 | -0.3 | 0.2 | 0.0 | 0.2 | 0.1 | 0.1 | -0.1 | 0.1 |
| V | 0.0 | 0.0 | -0.2 | -0.1 | -0.2 | -0.3 | 0.0 | 0.3 | -0.1 | 0.3 | 0.0 | 0.4 | 0.1 | 0.3 | -0.1 | -0.1 | 0.2 | 0.1 | 0.2 | 0.5 | 0.6 | | 0.3 | 0.1 | 0.4 | 0.2 | 0.5 | 0.6 | 0.1 | 0.5 | 0.4 | 0.3 | -0.2 | 0.3 | 0.1 | 0.3 | 0.3 | 0.2 | 0.0 | 0.2 |
| Ti | 0.2 | -0.6 | 0.0 | 0.3 | 0.1 | 0.3 | 0.1 | 0.0 | -0.1 | -0.1 | 0.1 | 0.3 | 0.3 | 0.4 | 0.5 | 0.4 | 0.5 | 0.5 | 0.5 | 0.5 | 0.4 | 0.3 | | 0.5 | 0.3 | 0.3 | 0.4 | 0.3 | 0.0 | 0.2 | 0.0 | 0.2 | -0.1 | 0.1 | -0.2 | 0.1 | 0.2 | -0.2 | -0.2 | 0.1 |
| Rb | 0.2 | -0.7 | 0.1 | 0.4 | 0.2 | 0.4 | -0.1 | 0.2 | -0.2 | 0.0 | 0.0 | 0.1 | 0.5 | 0.4 | 0.8 | 0.6 | 0.8 | 0.8 | 0.9 | 0.5 | 0.4 | 0.1 | 0.5 | | 0.2 | 0.7 | 0.1 | 0.2 | -0.2 | 0.0 | -0.2 | -0.1 | -0.2 | -0.2 | -0.3 | -0.2 | -0.2 | -0.3 | -0.4 | -0.3 |
| Sr | 0.1 | -0.2 | -0.1 | 0.0 | 0.0 | 0.1 | 0.3 | 0.4 | -0.3 | -0.1 | -0.1 | 0.1 | 0.3 | 0.8 | 0.2 | 0.3 | 0.3 | 0.3 | 0.3 | 0.7 | 0.6 | 0.4 | 0.3 | 0.2 | | 0.0 | 0.4 | 0.6 | 0.0 | 0.3 | 0.3 | 0.1 | -0.3 | 0.1 | -0.1 | 0.1 | 0.0 | 0.0 | -0.2 | 0.0 |
| Tl | -0.1 | -0.3 | 0.1 | 0.2 | 0.1 | 0.1 | -0.4 | 0.3 | 0.0 | 0.2 | 0.0 | 0.1 | 0.5 | 0.4 | 0.4 | 0.3 | 0.5 | 0.5 | 0.5 | 0.4 | 0.3 | 0.2 | 0.3 | 0.7 | 0.0 | | 0.0 | 0.1 | -0.2 | 0.0 | -0.1 | -0.2 | -0.2 | -0.2 | -0.2 | -0.3 | 0.0 | -0.2 | -0.4 | -0.3 |
| La | 0.2 | -0.1 | -0.1 | 0.0 | -0.1 | 0.1 | 0.3 | 0.1 | -0.2 | -0.1 | 0.1 | 0.3 | 0.2 | 0.2 | 0.1 | 0.2 | 0.2 | 0.1 | 0.2 | 0.3 | 0.5 | 0.5 | 0.4 | 0.1 | 0.4 | 0.0 | | 0.6 | 0.4 | 0.5 | 0.2 | 0.6 | -0.2 | 0.4 | -0.1 | 0.4 | 0.3 | 0.1 | 0.0 | 0.3 |
| Ba | 0.0 | -0.1 | -0.1 | 0.0 | 0.0 | 0.0 | 0.2 | 0.3 | -0.2 | 0.0 | 0.1 | 0.3 | 0.3 | 0.5 | 0.0 | 0.2 | 0.3 | 0.2 | 0.2 | 0.4 | 0.6 | 0.6 | 0.3 | 0.2 | 0.6 | 0.1 | 0.6 | | 0.3 | 0.5 | 0.4 | 0.3 | -0.3 | 0.3 | -0.1 | 0.3 | 0.2 | 0.1 | -0.1 | 0.2 |
| Cd | 0.2 | 0.0 | -0.1 | -0.1 | -0.1 | 0.0 | 0.0 | -0.1 | 0.0 | -0.1 | 0.1 | 0.3 | 0.1 | -0.1 | -0.1 | 0.1 | -0.2 | -0.2 | -0.1 | -0.1 | 0.1 | 0.1 | 0.0 | -0.2 | 0.0 | -0.2 | 0.4 | 0.3 | | 0.3 | 0.1 | 0.4 | -0.1 | 0.4 | 0.2 | 0.4 | 0.3 | 0.2 | 0.2 | 0.3 |
| U | 0.0 | 0.0 | -0.2 | -0.1 | -0.2 | -0.1 | 0.1 | -0.1 | -0.3 | 0.0 | -0.1 | 0.1 | -0.1 | 0.1 | 0.0 | -0.1 | 0.1 | 0.1 | 0.1 | 0.2 | 0.3 | 0.5 | 0.2 | 0.0 | 0.3 | 0.0 | 0.5 | 0.5 | 0.3 | | 0.7 | 0.6 | 0.2 | 0.7 | 0.3 | 0.7 | 0.5 | 0.5 | 0.4 | 0.7 |
| Te | 0.0 | 0.1 | -0.1 | -0.1 | -0.1 | -0.2 | 0.1 | 0.0 | -0.2 | 0.1 | 0.0 | 0.0 | -0.2 | 0.1 | -0.2 | -0.2 | -0.1 | -0.2 | -0.1 | 0.0 | 0.3 | 0.4 | 0.0 | -0.2 | 0.3 | -0.1 | 0.2 | 0.4 | 0.1 | 0.7 | | 0.5 | 0.3 | 0.5 | 0.5 | 0.6 | 0.4 | 0.6 | 0.5 | 0.6 |
| Ce | 0.1 | 0.0 | -0.1 | -0.1 | -0.1 | 0.0 | 0.2 | -0.2 | -0.2 | 0.0 | 0.0 | 0.0 | -0.1 | -0.1 | 0.0 | 0.0 | -0.1 | -0.1 | 0.0 | -0.1 | 0.2 | 0.3 | 0.2 | -0.1 | 0.1 | -0.2 | 0.6 | 0.3 | 0.4 | 0.6 | 0.5 | | 0.3 | 0.9 | 0.4 | 0.9 | 0.4 | 0.4 | 0.5 | 0.8 |
| B | -0.1 | 0.1 | 0.1 | 0.0 | 0.0 | 0.0 | -0.1 | -0.3 | 0.1 | 0.1 | -0.2 | -0.3 | -0.3 | -0.2 | -0.1 | -0.2 | -0.3 | -0.1 | -0.2 | -0.3 | -0.3 | -0.2 | -0.1 | -0.2 | -0.3 | -0.2 | -0.2 | -0.3 | -0.1 | 0.2 | 0.3 | 0.3 | | 0.4 | 0.6 | 0.4 | 0.2 | 0.3 | 0.8 | 0.5 |
| Pr | 0.1 | 0.1 | -0.1 | -0.1 | -0.1 | 0.0 | 0.1 | -0.2 | -0.2 | 0.0 | -0.1 | 0.0 | -0.2 | -0.1 | -0.1 | -0.1 | -0.1 | -0.1 | -0.1 | -0.1 | 0.2 | 0.3 | 0.1 | -0.2 | 0.1 | -0.2 | 0.4 | 0.3 | 0.4 | 0.7 | 0.5 | 0.9 | 0.4 | | 0.5 | 0.9 | 0.4 | 0.5 | 0.6 | 0.9 |
| Be | -0.1 | 0.2 | -0.2 | -0.3 | -0.3 | -0.2 | 0.0 | -0.1 | 0.0 | 0.2 | -0.1 | -0.1 | -0.4 | -0.3 | -0.3 | -0.3 | -0.3 | -0.3 | -0.2 | -0.3 | 0.0 | 0.1 | -0.2 | -0.3 | -0.1 | -0.2 | -0.1 | -0.1 | 0.2 | 0.3 | 0.5 | 0.4 | 0.6 | 0.5 | | 0.5 | 0.2 | 0.5 | 0.8 | 0.6 |
| Nd | 0.0 | 0.1 | -0.1 | -0.1 | -0.1 | -0.1 | 0.1 | -0.2 | -0.3 | 0.0 | -0.1 | 0.0 | -0.2 | -0.1 | -0.1 | -0.1 | -0.1 | -0.1 | -0.1 | -0.1 | 0.2 | 0.3 | 0.1 | -0.2 | 0.1 | -0.3 | 0.4 | 0.3 | 0.4 | 0.7 | 0.6 | 0.9 | 0.4 | 0.9 | 0.5 | | 0.5 | 0.6 | 0.7 | 0.9 |
| Pb | -0.3 | 0.2 | -0.1 | -0.1 | -0.1 | -0.3 | -0.1 | -0.2 | 0.1 | 0.0 | 0.1 | 0.1 | 0.0 | -0.1 | -0.2 | -0.1 | -0.1 | -0.2 | -0.1 | -0.1 | 0.1 | 0.3 | 0.2 | -0.2 | 0.0 | 0.0 | 0.3 | 0.2 | 0.3 | 0.5 | 0.4 | 0.4 | 0.2 | 0.4 | 0.2 | 0.5 | | 0.5 | 0.3 | 0.5 |
| Pt | -0.1 | 0.2 | -0.1 | -0.1 | -0.2 | -0.1 | 0.0 | -0.3 | -0.1 | 0.1 | 0.1 | 0.0 | -0.2 | -0.1 | -0.2 | -0.3 | -0.3 | -0.4 | -0.3 | -0.2 | 0.1 | 0.2 | -0.2 | -0.3 | 0.0 | -0.2 | 0.1 | 0.1 | 0.2 | 0.5 | 0.6 | 0.4 | 0.3 | 0.5 | 0.5 | 0.6 | 0.5 | | 0.5 | 0.6 |
| Li | -0.1 | 0.2 | -0.1 | -0.2 | -0.2 | -0.2 | 0.0 | -0.2 | -0.1 | 0.0 | -0.1 | -0.2 | -0.4 | -0.3 | -0.3 | -0.3 | -0.3 | -0.3 | -0.3 | -0.4 | -0.1 | 0.0 | -0.2 | -0.4 | -0.2 | -0.4 | 0.0 | -0.1 | 0.2 | 0.4 | 0.5 | 0.5 | 0.8 | 0.6 | 0.8 | 0.7 | 0.3 | 0.5 | | 0.7 |
| Sa | 0.0 | 0.1 | 0.0 | -0.1 | -0.1 | -0.1 | 0.1 | -0.3 | -0.2 | 0.0 | 0.0 | 0.0 | -0.2 | -0.2 | -0.2 | -0.1 | -0.2 | -0.2 | -0.2 | -0.2 | 0.1 | 0.2 | 0.1 | -0.3 | 0.0 | -0.3 | 0.3 | 0.2 | 0.3 | 0.7 | 0.6 | 0.8 | 0.5 | 0.9 | 0.6 | 0.9 | 0.5 | 0.6 | 0.7 | |

**Supplemental Figure S1**: Correlation table of Spearman's coefficient ϱ values for PCBs and inorganic elements having 5 or more detectable values. Green variables are of interest, yellow are nutritional elements, and grey are elements with mostly (50 to 5) "non-detects" (grey elements ordered from

highest to lower number of "non-detects"). Red shades indicate positive correlations, and blue shades indicate negative correlations. Pink/light blue is for p = 0.05 to 0.01 (ϱ = 0.195 to 0.255); medium pink/medium blue is for $p$ = 0.01 to 0.001 (ϱ = 0.255 to 0.324); red/dark blue is for $p$ = 0.001 to 0 (ϱ = 0.324 to 1); black is for ϱ = 1; white for no statistically significant correlation.

| Var | Age | Hg | As | Ca | Cr | Co | Cu | Fe | Mg | Mn | P | K | Na | S | Se | V | Zn |
|---|---|---|---|---|---|---|---|---|---|---|---|---|---|---|---|---|---|
| Age | | 0.6 | 0.4 | 0.0 | -0.2 | 0.1 | -0.2 | 0.1 | 0.0 | 0.0 | 0.0 | 0.1 | 0.3 | 0.2 | -0.1 | 0.0 | 0.0 |
| Hg | 0.6 | | 0.2 | 0.1 | -0.3 | 0.0 | -0.3 | 0.0 | 0.3 | 0.2 | 0.3 | 0.3 | 0.5 | 0.5 | 0.0 | -0.3 | 0.2 |
| As | 0.4 | 0.2 | | -0.1 | -0.3 | 0.0 | -0.1 | 0.0 | 0.0 | -0.1 | -0.1 | 0.1 | 0.3 | 0.1 | 0.3 | -0.1 | -0.1 |
| Ca | 0.0 | 0.1 | -0.1 | | 0.0 | 0.4 | -0.1 | 0.1 | 0.4 | 0.7 | 0.6 | 0.2 | 0.4 | 0.2 | -0.2 | 0.4 | 0.6 |
| Cr | -0.2 | -0.3 | -0.3 | 0.0 | | 0.1 | 0.3 | 0.4 | 0.0 | 0.0 | 0.0 | -0.1 | -0.2 | -0.1 | 0.2 | 0.3 | 0.0 |
| Co | 0.1 | 0.0 | 0.0 | 0.4 | 0.1 | | 0.0 | 0.4 | 0.3 | 0.5 | 0.3 | 0.2 | 0.3 | 0.1 | -0.1 | 0.7 | 0.4 |
| Cu | -0.2 | -0.3 | -0.1 | -0.1 | 0.3 | 0.0 | | 0.5 | -0.2 | -0.1 | -0.1 | -0.2 | -0.1 | -0.1 | 0.3 | 0.0 | 0.0 |
| Fe | 0.1 | 0.0 | 0.0 | 0.1 | 0.4 | 0.4 | 0.5 | | 0.0 | 0.3 | 0.2 | 0.0 | 0.3 | 0.2 | 0.2 | 0.4 | 0.3 |
| Mg | 0.0 | 0.3 | 0.0 | 0.4 | 0.0 | 0.3 | -0.2 | 0.0 | | 0.6 | 0.8 | 0.9 | 0.4 | 0.7 | 0.1 | 0.1 | 0.4 |
| Mn | 0.0 | 0.2 | -0.1 | 0.7 | 0.0 | 0.5 | -0.1 | 0.3 | 0.6 | | 0.6 | 0.5 | 0.4 | 0.4 | -0.2 | 0.4 | 0.6 |
| P | 0.0 | 0.3 | -0.1 | 0.6 | 0.0 | 0.3 | -0.1 | 0.2 | 0.8 | 0.6 | | 0.7 | 0.5 | 0.7 | 0.0 | 0.2 | 0.5 |
| K | 0.1 | 0.3 | 0.1 | 0.2 | -0.1 | 0.2 | -0.2 | 0.0 | 0.9 | 0.5 | 0.7 | | 0.4 | 0.7 | 0.1 | 0.1 | 0.4 |
| Na | 0.3 | 0.5 | 0.3 | 0.4 | -0.2 | 0.3 | -0.1 | 0.3 | 0.4 | 0.4 | 0.5 | 0.4 | | 0.6 | 0.0 | 0.0 | 0.5 |
| S | 0.2 | 0.5 | 0.1 | 0.2 | -0.1 | 0.1 | -0.1 | 0.2 | 0.7 | 0.4 | 0.7 | 0.7 | 0.6 | | 0.2 | -0.2 | 0.4 |
| Se | -0.1 | 0.0 | 0.3 | -0.2 | 0.2 | -0.1 | 0.3 | 0.2 | 0.1 | -0.2 | 0.0 | 0.1 | 0.0 | 0.2 | | -0.1 | 0.0 |
| V | 0.0 | -0.3 | -0.1 | 0.4 | 0.3 | 0.7 | 0.0 | 0.4 | 0.1 | 0.4 | 0.2 | 0.1 | 0.0 | -0.2 | -0.1 | | 0.2 |
| Zn | 0.0 | 0.2 | -0.1 | 0.6 | 0.0 | 0.4 | 0.0 | 0.3 | 0.4 | 0.6 | 0.5 | 0.4 | 0.5 | 0.4 | 0.0 | 0.2 | |

**Supplemental Figure S2:** Correlation table of Spearman's coefficient ϱ values for selected inorganic elements. Elements were chosen based on correlations seen for Hg and As in PCB correlation analysis (Supplemental Figure S1). All elements were detected in most samples, except V (57% detectable). Pink/light blue is for $p$ = 0.05 to 0.01 (ϱ = 0.084 to 0.11); medium pink/medium blue is for $p$ = 0.01 to 0.001 (ϱ = 0.11 to 0.0.143); red/dark blue is for $p$ = 0.001 to 0 (ϱ = 0.143 to 1); black is for ϱ = 1; white for no statistically significant correlation.

**Principal Components Analysis (PCA)**

Samples were examined with principal components analysis (PCA) to explore whether any differences between variables such as fish species, sampling location, or year could be discerned. PCA is a multivariate statistical technique that can be used to "fingerprint" samples based on relevant descriptive variables, like concentrations of inorganic elements or concentrations/proportions of PCB congeners. PCA is generally used as an exploratory or descriptive tool. PCA is used when more than two or three variables represent the data because looking for relationships or patterns using a graphical representation in four, or greater, dimensional space would be too difficult. The positions of samples on a reduced

(usually two or three dimensional) plot, which has axes that are linear combinations of the original n variables, can be examined to ascertain similarities between samples. The analysis is based on the assumption that some of the variables may be correlated, which was demonstrated in the previous section (correlation analysis). PCA reduces the dataset to capture the maximum possible variability in the data.

Many variables in the present study are dominated by "non-detect" values. "Poor performance" of PCA was identified in a groundwater study when non-detects were imposed on the datasets at a rate of approximately 20 to 35% [91]. Nevertheless, others have conducted PCA on groundwater data with higher non-detect rates (*e.g.* up to 46% non-detects) [92]. For the PCA of total PCB variables with other variables (inorganic elements, age and % lipid), referred to as PCA-all, variables with fewer than 70% detectable values were excluded, except for Cr and vanadium (V), which were detected in 64 and 66 samples respectively. These elements were included because they were close to the 70% detectable criterion and because including variables with these rates of non-detects was not inconsistent with methods and findings in previous similar studies [91, 92]. For the PCA of PCB congeners (PCA-congeners), all variables with 5 or more detectable values were included because the low occurrence of some congeners in the fish was considered a valuable characteristic to include in the analysis.

Substitutions for <LOD values were obtained as log-normal LROS values (using ProUCL 5.0) [93]. Substitutions are made in ROS by fitting a regression line to the normal scores of the order statistics for the detectable observations and then filling in values imputed from the straight line for the observations below the detection limit [93].

Two PCAs were carried out for PCA-all: one using untransformed data and the other using log-transformed data. The results were similar and the PCA results for the log-transformed data will be discussed in more detail as the samples were better visualized. Three PCAs were carried out for PCA-congeners: one using untransformed concentrations data, one using log-transformed concentration data, and one using untransformed proportions (fraction of each congener of the sum). The results were similar, and in this case, the PCA results for the proportions data were better visualized and will be discussed in more detail.

In the PCA-all analysis, each sample location was represented by 25 variables, and in the PCB-congeners, the number of variables was 85. The analysis was based on the assumption that some of the variables may be correlated. PCA reduces the dataset to capture the maximum possible variability in the data. One PCA result was the generation of new variables (factors) numbering the same as the original variables (25 for PCA-all and 85 for PCA-congeners). The number of significant factors according to the Kaiser criterion, which states that factors with eigenvalues (a measure of the variability) greater than one are significant, is 9 and 10 for PCA-all, and 9–13 for PCA-congeners. For the purposes of keeping the data interpretation simple and easy to visualize, only the first two factors will be further considered. The corresponding percent variance for the first two factors generated for PCA-all was 29% for Factor 1 and 15% for Factor 2, giving a sum of 44%. For PCB congeners, the percent variance for Factor 1 was 32% and for Factor 2, 14%, giving a sum of 46% Less than half of the variance, which can also be thought of the original information in the total dataset, is thus represented by the new factors for both PCAs. This representation and the interpretation of the resulting groupings should be considered preliminary, with findings considered to be of an exploratory nature only.

***PCA-all***. The factors for each variable are plotted in two dimensions in Figure S3A. This graphical representation reveals the influence of the original variables on the PCA. Hg and the PCB variables plot close together (indicating correlations, Pearson r = 0.25–0.63) in the lower right quadrant (positive on Factor 1, negative on Factor 2); As and Se plot fairly close together; the nutritional elements plot positively on Factor 1; and Cu and % lipids plot negatively on Factor 1 (indicating negative correlations with other elements).

Factors 1 and 2 for each sample were also plotted (Figure S3B) with symbols representing fish species (same symbol) and groups (same colour group of blue, red, yellow or green). Generally the samples plot together according to fish species, with Arctic char (diamonds) and lake trout (circles) overlapping substantially and plotting generally along the negative x-axis, Factor 1) but a few lake trout outliers found in the positive x-axis side of the plot. Lake whitefish (squares) generally plot slightly more to the positive end of Factor 1 and overlap with cisco (triangles).

Group 1 lake whitefish and group 4 cisco appear to plot differently from each other (albeit reflected by only three samples each). Examination of the variable concentrations in these fish samples revealed that while many were the same (Ca, Cr, Fe, Mn, Ni, P, Ti), and the fish were matched for size (length and size) and % lipid, some variable concentrations differed. Specifically, PCBs, B, Rb, Se, Na, S, Tl, Zn, and Hg concentrations were higher in group 1 lake whitefish, and As, Ba, Co, Mg, K, Sr, and V concentrations were higher in group 4 cisco. The reasons for higher concentrations of the monovalent ionic elements Rb, Na, and Tl in site 1 lake whitefish and divalent ionic elements Ba, Mg and Sr (but also monovalent K) are unknown, with the possibility that they could be related to fish biology and/or site geology. This could be an intriguing area to investigate.

The two fish with Hg exceedances plot in the lower right quadrant, along with one of the fish samples that exceed the PCB6 (EU) guideline [94] and the three fish with the highest As concentrations. The other fish sample that exceeds the PCB6 (EU) guideline [94] is also in the bottom half of the plot, but close to the y-axis.

***PCA-congeners***. The factors for each variable are plotted in two dimensions in Figure S4A. The number of chlorines on the PCB congener is indicated by colour. Congeners with 5 and 6 chlorines dominate the bottom half of the plot, whereas the congeners with a low (3 and 4 chlorines) and high (7, 8 and 9 chlorines) degrees of chlorination dominate the top right corner, although much scatter is seen in all groups. The major congeners found in PCB Aroclor mixtures are indicated by circles (pink for 1254 and orange for 1260; major congeners for other mixtures are not present or only a few are present). Factors 1 and 2 for each sample were also plotted (Figure S4B) with symbols as for Figure S3B. Samples of the same fish species appear to be spread out, mostly along Factor 2, with a narrower range along Factor 1 for most species. Char from geographic group 2 appear to be different from other char, plotting more to the right (along Factor 1). The two fish with PCB6 exceedances are again circled in pink, and the group 2 char that plots somewhat apart from the other fish in this group contains the highest total PCB concentration (more than double the next highest PCB concentration) and is a PCB6 exceedance.

The three group 1 lake whitefish samples appear to plot together in the lower left quadrant, approximately at the extreme end of the cluster that is made up of most of the samples. The majority of samples (51 of 101) were found in the centre of the plot, between -4.5 and 4.5 on the x-axis (Factor 1) and -3 and 3 on the y-axis (Factor 2).

To further investigate any differences in congener profiles in the fish samples, bar graphs of the congener proportions (Figure S5) and line graphs (Figure S6) were constructed. The information in Figures S5 and S6 is the same; these figures differ only in their presentation. In Figure S5 and S6 the congener profiles, using averages of samples closest together in the PCA groups, are shown. Char from geographic group 1 and group 2, lake whitefish from group 1, the central group (representing 51% of samples), and Aroclor 1254 and 1260 profiles are shown. The profiles do not appear to be dramatically different, with several congeners predominating in most samples and PCB 153, 118, and 138 as the largest. All three of these congeners also predominate in Aroclor 1260 and are used in environmental samples (*e.g.* soil) as indicative congeners in identifying this PCB mixture. PCB 118 also occurs in Aroclor 1254, along with another predominating peak, PCB 110. These findings suggest that the fish have retained some of the characteristic peaks of these two Aroclor mixtures.

1-Char (char from geographic group 1) appeared to have higher proportions of low and high chlorinated PCB congeners (to the extreme left and right on the x-axis), and more congeners present

overall (resulting in lower proportions of all congeners, including the dominant ones). 2-Char (from geographic group 2) was similar to 1-Char with the presence of low and high chlorinated PCBs but had larger amounts of the dominating congeners. The low and high chlorinated congeners appeared to be nearly absent in 1-lake whitefish (1-LWF). The proportions of dominant peaks were much higher in the central group, showing the common predominance of the PCB congeners (in descending order of proportions) 153, 118, 138, 110, 99, 101, and 102+93+98+95.

**A.**

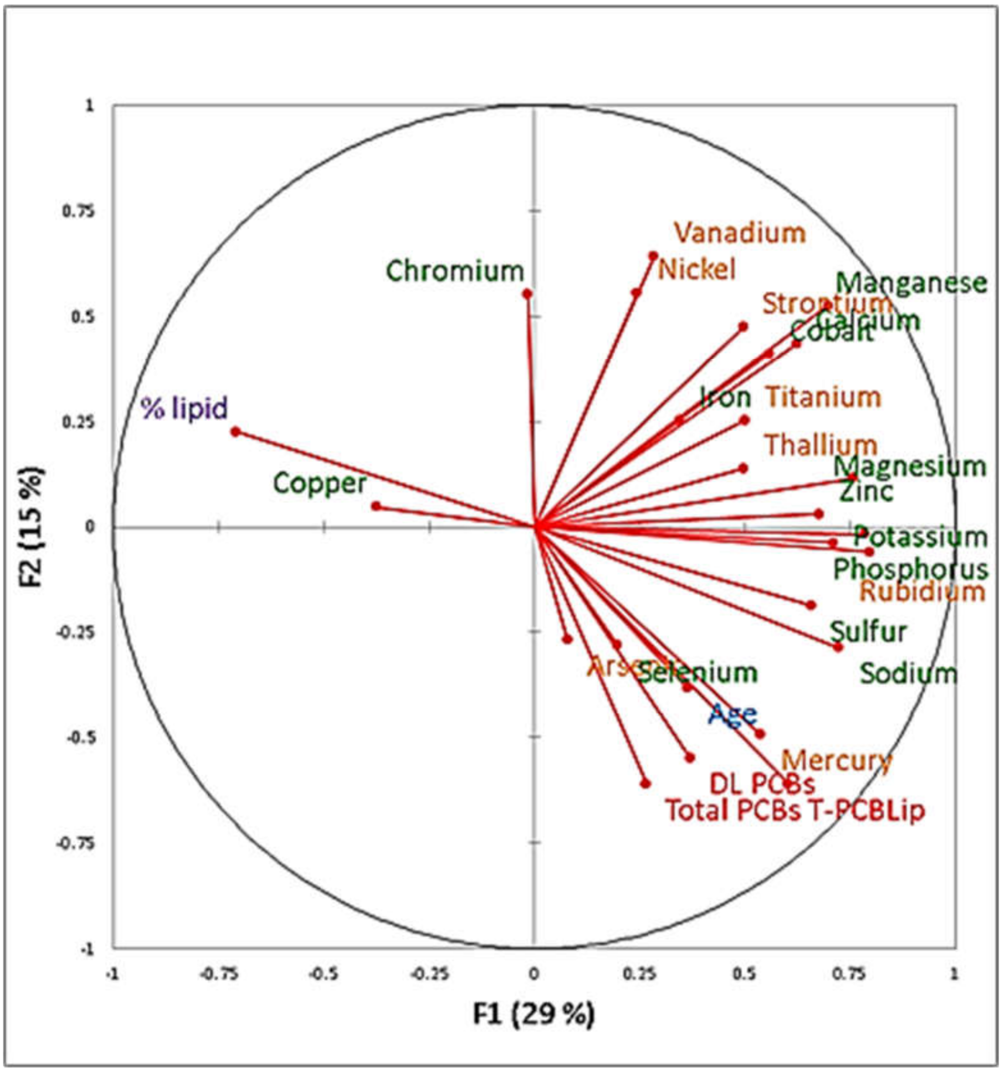

**B.**

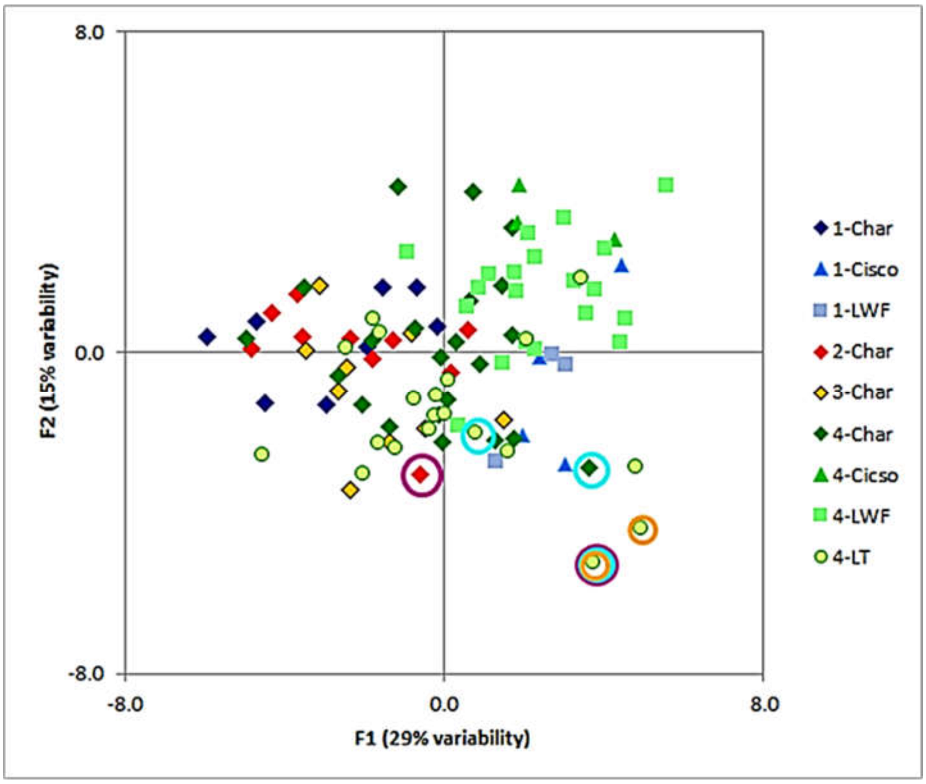


**Supplemental Figure S3: A.** Factor loadings of variables used in PCA for all variables (PCBs, inorganic elements, age and % lipid). Variables are colour coded as: blue/purple = age/lipid; green = nutritional elements; orange = other/contaminant elements; red = PCBs. **B.** Factor loadings of samples used in PCA for all variables. Pink circles are samples that exceeded the EU guideline for PCB6 [94]; orange circles are Hg exceedances; Blue circles have highest As (>100 mg/kg). Fish samples indicated are identified as to their geographic grouping such as 1-Char (Arctic char in geographic group 1 etc.).

**A.**

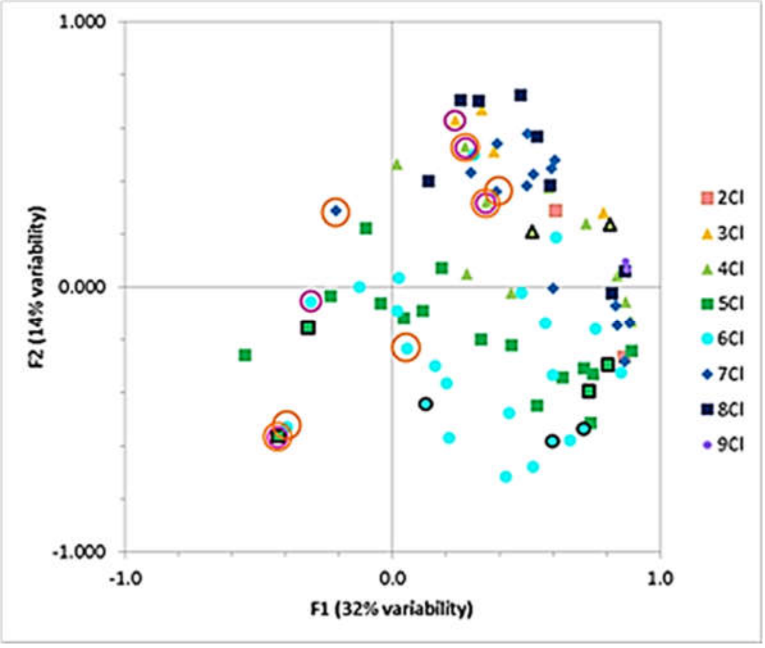


**B.**

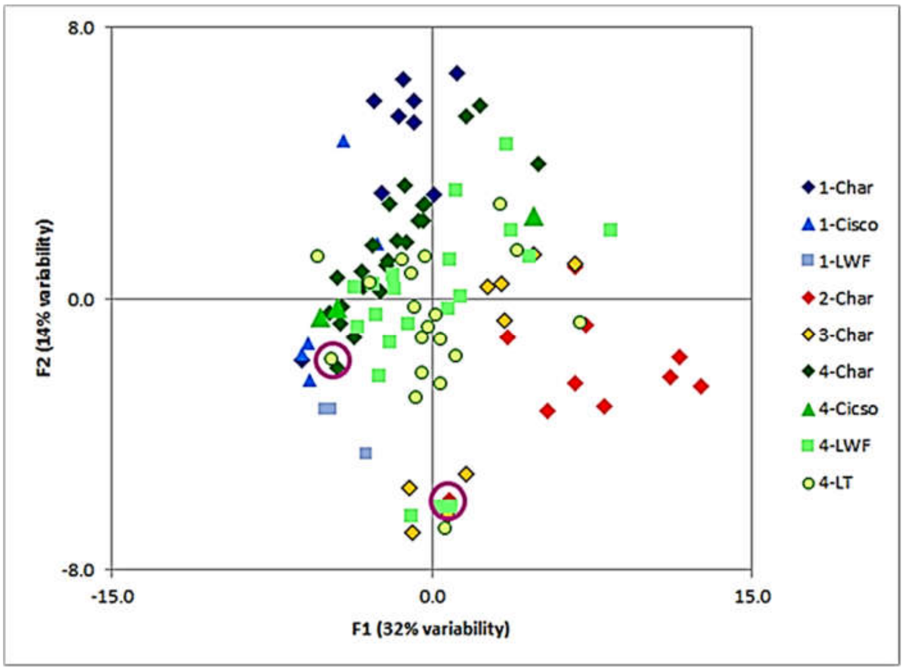


**Supplemental Figure S4: A.** Factor loadings of variables used in PCA for PCB congeners. Pink circled congeners are characteristic of Aroclor 1254 and orange for 1260. Black outline indicates DL-PCBs. **B.** Factor loadings of samples used in PCA for PCB congeners. Pink circles are samples that exceeded the EU guideline for PCB6 [94]. Fish samples indicated are identified as to their geographic grouping such as 1-Char (Arctic char in geographic group 1 etc.)

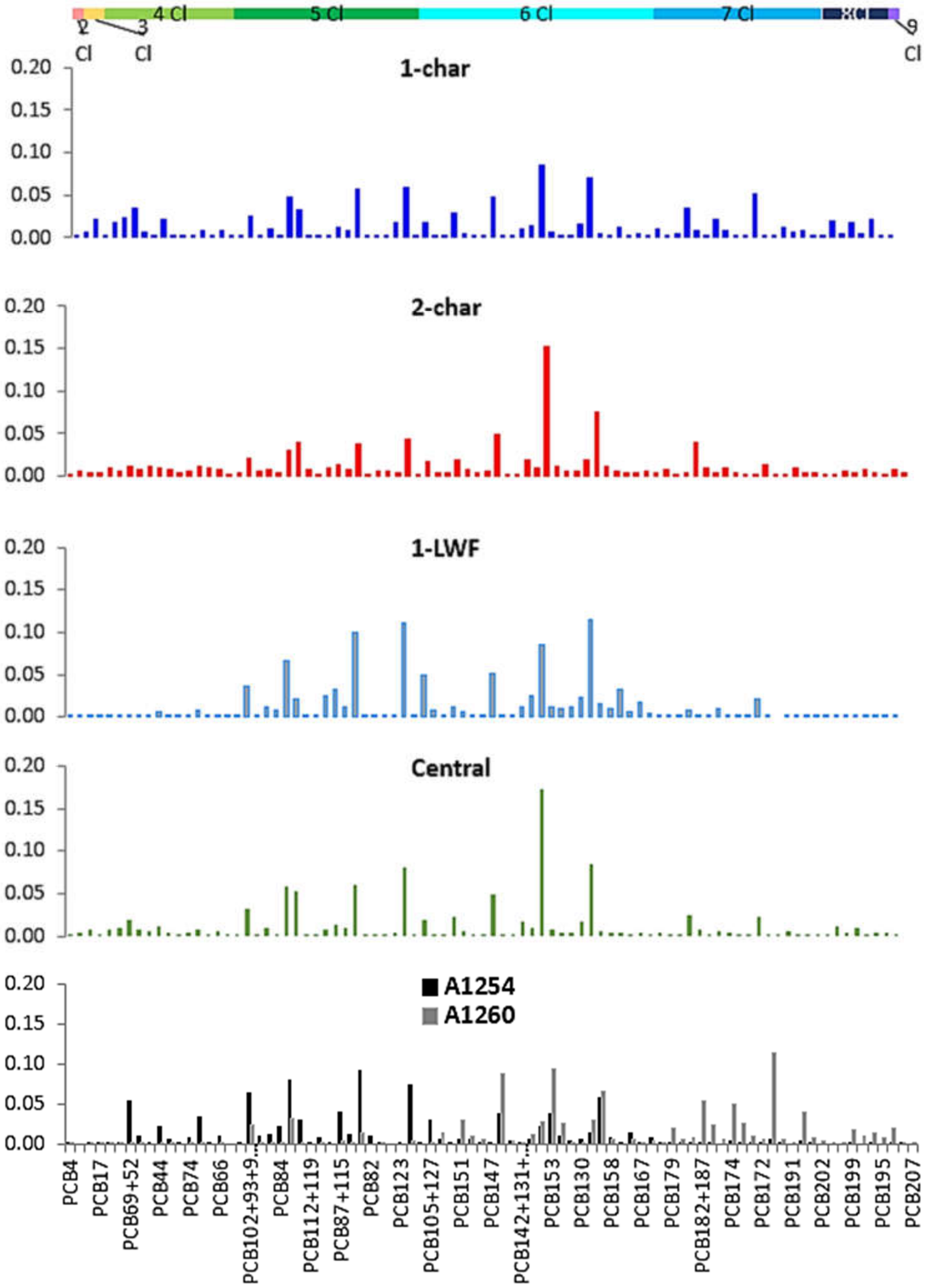


**Supplemental Figure S5:** Proportions (unitless) of PCB congeners in groups from PCA (1-char = char from group 1, 2-char = char from group 2, 1-LWF = lake whitefish from group 1, central = fish from center of PCA) and Aroclor 1254 (A1254) and 1260 (A1260). Proportions are shown on the same scale (maximum 0.2 = 20%) to allow comparisons and the number of chlorines in the PCB molecules are shown in upper bar.

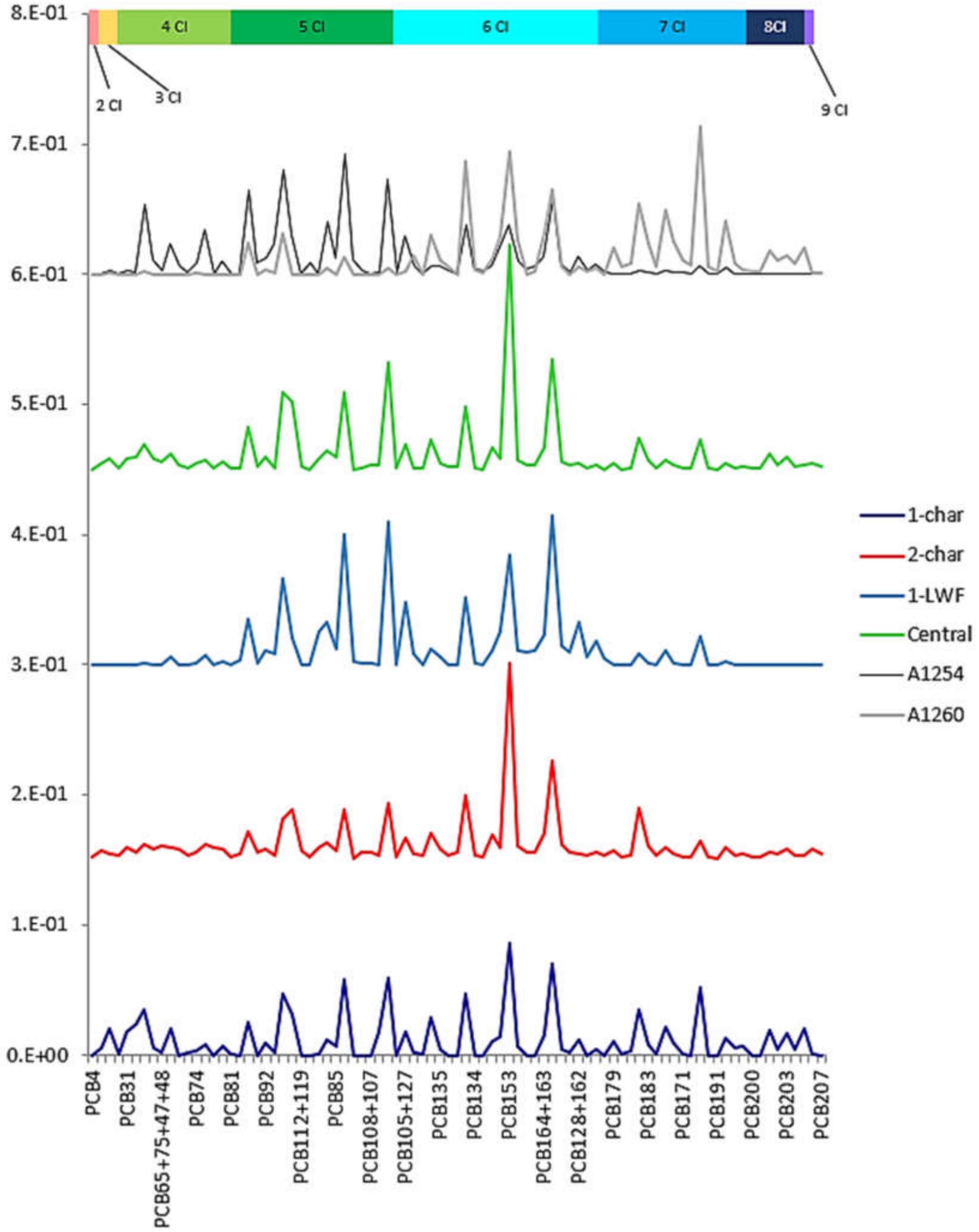


**Supplemental Figure S6.** Proportions (unitless) of PCB congeners as line graphs in groups from PCA (1-char = char from group 1, 2-char = char from group 2, 1-LWF = lake whitefish from group 1, central = fish from centre of PCA) and Aroclor 1254 (A1254) and 1260 (A1260). Graphs are offset by a proportion value of 0.15 to allow for comparisons, different profiles as indicated in the legend, and the number of chlorines in the PCB molecules are shown in upper bar.

**A.**

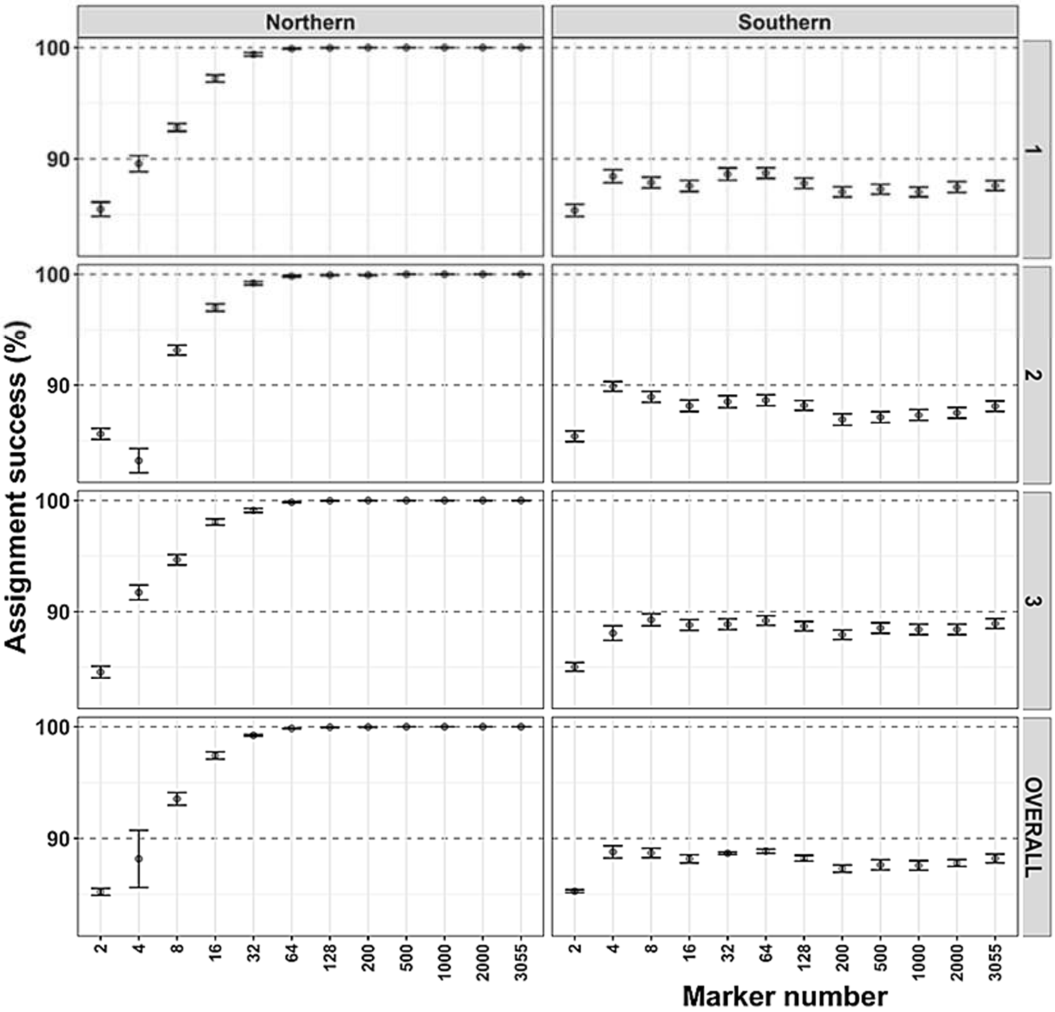

Northern
Southern
1
2
3
OVERALL
100
90
Assignment success (%)
2
4
8
16
32
64
128
200
500
1000
2000
3055
Marker number

B.

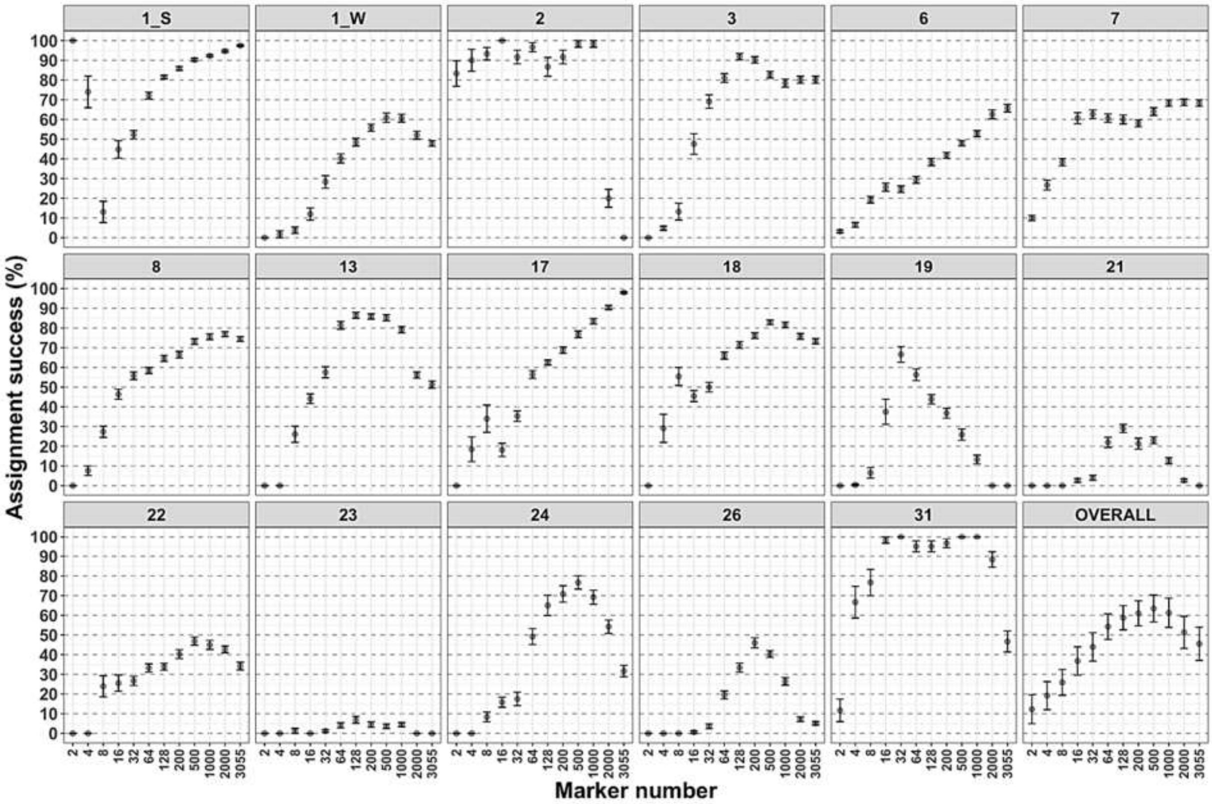


**Supplemental Figure S7:** Arctic char single nucleotide polymorphisms or markers used for assignments with success rates shown as a function of the number of markers used but with missing data imputed (similar to Fig. 4 where data was not imputed). **A**. Assignment tests to Northern or Southern populations **B**. Fishing site assignment tests, with fishing sites listed with their numbers as indicated in Table 1.

**A.**

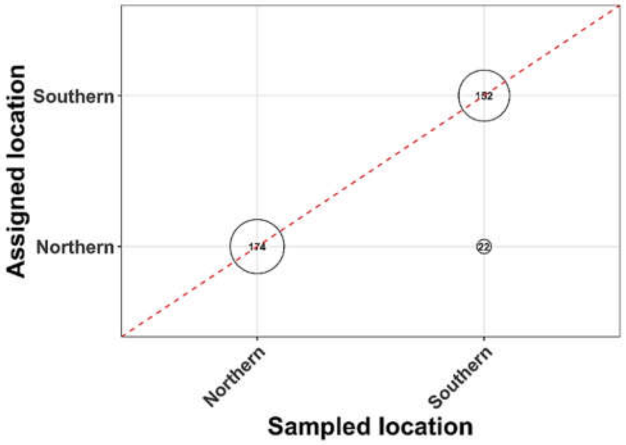


**B.**

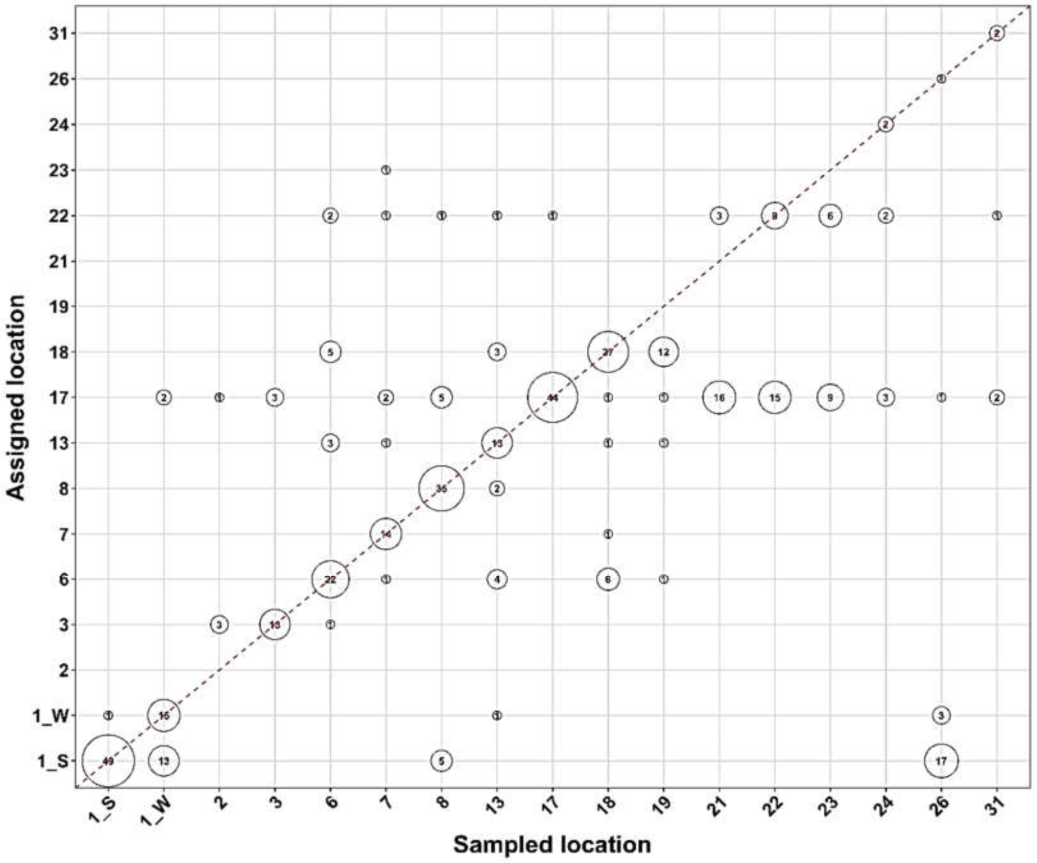


**Supplemental Figure S8**: Assignment results with Arctic char single nucleotide polymorphism markers (N=3055) with missing data imputed. The circles on the red dashed diagonal represent successful assignments, and other circles display mis-assignments in which individuals were not successfully assigned. Circle sizes are proportional to the number of individuals. **A.** Regional-level assignment to Northern or Southern fish populations with missing data imputed. **B.** Fishing site l assignment tests with missing data imputed with fishing sites listed as in Table 1 (site 1 is shown as fish caught in spring and winter as 1_S and 1_W, respectively).

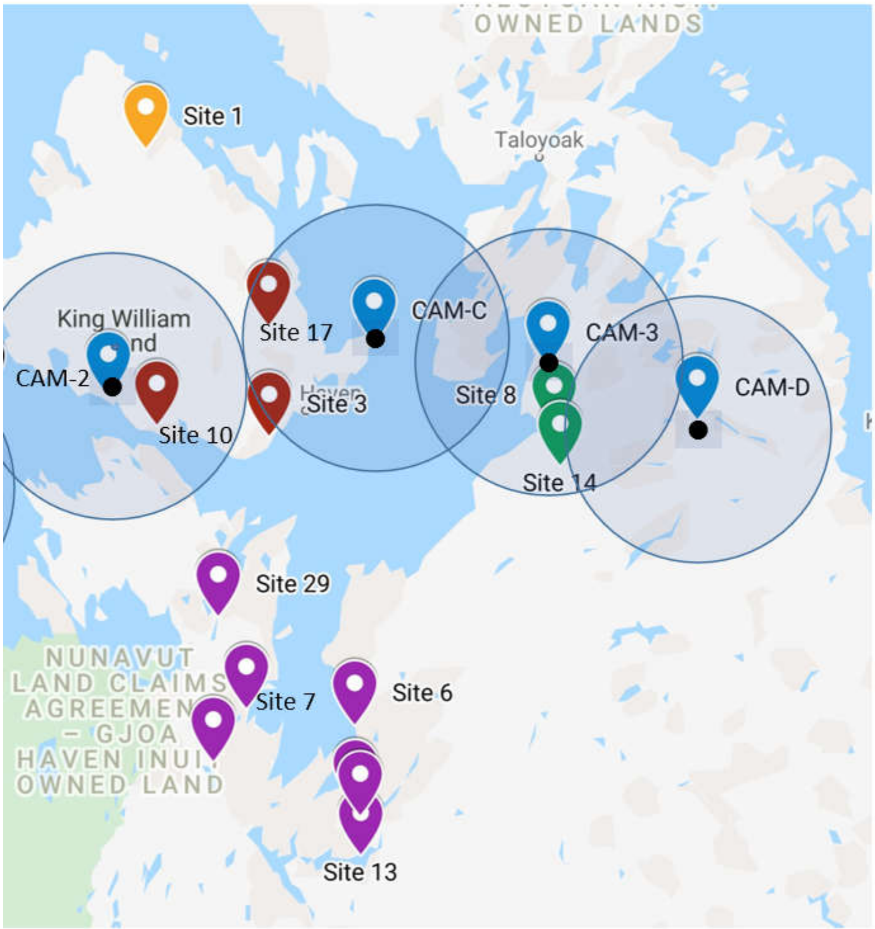


**Supplementary Figure S9.** Map of the Distant Early Warning stations (DEW line) in this region of the Arctic. Shown are each station (blue pins) and their site codes with grey 50 km halos around each. From west to east CAM-2 and CAM-3 are at Gladman Point and Matheson Point on King William Island, respectively, with CAM-3 at Shepherd Bay and CAM-D at Simpson Lake both situated on the mainland to the east. Selected fishing sites are shown as pins (orange for group 1, brown for group 2, green for group 3 and purple for group 4, as indicated in Fig. 1), with a few of these numbered for convenience.

**Supplemental Tables:**

**Supplemental Table S1**

Details of calculated PCB parameters

| CALCULATED PARAMETER | CALCULATION DETAILS | |
|---|---|---|
| Total PCBs | Sum of all congeners, not including non-detects (which were given a value of zero) | |
| Total PCBs on a lipid weight basis (T-PCBlip) | Total PCB value divided by fraction lipid | |
| Dioxin-like (dl)-PCBS | Sum of dioxin-like PCB congeners, each multiplied by their respective toxic equivalence factor (TEF), summarized below: | |
| | **Congener #** | **TEF**[A] |
| | 77 | 0.0001 |
| | 81 | 0.0003 |
| | 126 | 0.1 |
| | 169 | 0.03 |
| | 105 | 0.00003 |
| | 114 | 0.00003 |
| | 118 | 0.00003 |
| | 123 | 0.00003 |
| | 156 | 0.00003 |
| | 157 | 0.00003 |
| | 167 | 0.00003 |
| | 189 | 0.00003 |
| Non-dioxin-like PCBS | Dioxin-like PCB congeners (not adjusted for TEF) subtracted from Total PCBs | |
| PCB6 (EU) | Sum of six non-dioxin-like PCB congeners defined by EU as indicator PCBs: PCB 28, 52, 101, 138, 153, 180 [94]. | |

[A] From Health Canada 2012 [95]

**Supplemental Table S2**

List of samples and concentrations (µg $kg^{-1}$ ww) that exceed the Ontario guideline [96] used for setting fish advisories for freshwater sport fish (26 µg $kg^{-1}$ ww), and exceedances of other available guidelines, noting that all total PCB concentrations are below the value for PCBs (2000 µg $kg^{-1}$ ww) listed in the formerly used "Canadian guidelines for chemical contaminants and toxins in fish and fish products" [A] .

| SAMPLE ID | YEAR | SPECIES | LENGTH (MM) | TOTAL PCBS[A] | DL-PCBS (TEQ)[B] | PCB6 (EU)[C] |
|---|---|---|---|---|---|---|
| *GUIDELINE* | | | | 26 | 2.0E-02 | 75 |
| **1-2218** | 2017 | Cisco | 320 | 58 | 3.4E-04 | 167 |
| **1-2224** | 2017 | Cisco | 320 | 42 | 2.5E-04 | 13 |
| **1-2225** | 2017 | Cisco | 323 | 27 | 1.7E-04 | 8.5 |
| **6-72** | 2016 | Lake trout | 760 | 45 | 1.8E-04 | 29 |
| **6-196** | 2016 | Lake trout | 895 | 140 | 2.1E-03 | 88 |
| **6-3107** | 2018 | Lake trout | 428 | 28 | 1.7E-04 | 9.9 |
| **7-487** | 2018 | Lake whitefish | 390 | 37 | 2.1E-04 | 13 |
| **7-498** | 2018 | Lake whitefish | 405 | 39 | 2.4E-04 | 15 |
| **8-77** | 2016 | Arctic char | 666 | 57 | 3.5E-04 | 23 |
| **8-971** | 2017 | Arctic char | 758 | 110 | 6.0E-04 | 41 |
| **8-1002** | 2017 | Arctic char | 620 | 40 | 2.0E-04 | 15 |
| **17-2713** | 2018 | Arctic char | 410 | 367 | 1.6E-02 | 101 |
| | | *AVERAGE (ALL FISH)* | | 15 | 2.5E-04 | 5.7 |

[A] Total PCBs = sum of all congeners measured. The guideline value is an Ontario value used to establish fish advisories in Ontario lakes and rivers [96].
[B] DL-PCBs = dioxin-like PCBs, which are corrected to a toxic equivalence value to 2,3,7,8-tetrachlorodibenzo-p-dioxin (TCDD). The guideline (for 2,3,7,8-TCDD) is listed in "List of Contaminants and other Adulterating Substances in Foods" [55].
[C] PCB6 (EU) = sum of 6 indicator non dioxin-like PCBs (PCB28, PCB52, PCB101, PCB138, PCB153 and PCB180) in fish (with the exemption of exclusively freshwater fish, for which a higher guideline exists) for the European Union [94].

**Supplemental Table S3**

Percent moisture for fish species showing percent moisture values from measurements performed at room temperature and used to convert dry weight Hg and PCB values to wet weight values.

| FISH SPECIES | FISH MEASURED (N) | MEAN % MOISTURE |
|---|---|---|
| Arctic char | 48 | 67 |
| Lake trout | 79 | 73 |
| Cisco | 72 | 69 |
| Lake whitefish | 37 | 68 |

**Supplemental References:**

**Cited in the main text and Supplement**


55. Health Canada 2000 and updated. Health Canada's Maximum Levels for Chemical Contaminants in Foods. Government of Canada. https://www.canada.ca/en/health-canada/services/food-nutrition/food-safety/chemical-contaminants/maximum-levels-chemical-contaminants-foods.html (accessed 12/8/2020).
72. Caumette, G.; Koch, I.; Reimer, K.J. Arsenobetaine formation in plankton: a review of studies at the base of the aquatic food chain. J. Environment. Monit., 2012, 14, 2841-2853. https://doi.org/10.1039/C2EM30572K


**Cited in the Supplement**


88. Pelletier, E. Mercury-selenium interactions in aquatic organisms: a review. Marine Environmental Research 1986, 18(2), 111–32. doi.org/10.1016/0141-1136(86)90003-6.
89. Bhattacharya, S.; Bhattacharya, A.; Roy, S. Arsenic-induced responses in freshwater teleosts. Fish Physiology and Biochemistry 2007, 33(4). 463–73. DOI 10.1007/s10695-007-9173-2. (11), 2841–53. DOI: 10.1039/c2em30572k.
90. Monteiro, D.A.; Rantin, F.T.; Kalinin, A.L. Inorganic mercury exposure: toxicological effects, oxidative stress biomarkers and bioaccumulation in the tropical freshwater fish matrinxã, *Brycon amazonicus* (Spix and Agassiz, 1829). Ecotoxicology 2010, 19(1), 105–123. doi.org/10.1007/s10646-009-0395-1
91. Farnham, I.M.; Singh, A.K.; Stetzenbach, K.J.; Johannesson, K.H. Treatment of nondetects in multivariate analysis of groundwater geochemistry data. Chemometrics and Intelligent Laboratory Systems 2002, 60(1–2, 28) 265-281. https://doi.org/10.1016/S0169-7439(01)00201-5
92. Olsen RL, Chappell RW, Loftis JC 2012. Water quality sample collection, data treatment and results presentation for principal components analysis – literature review and Illinois River watershed case study. Water Research 46 (9), 3110–3122 doi.org/10.1016/j.watres.2012.03.028.
93. U.S. EPA (United States Environmental Protection Agency) 2013. ProUCL 5.0.00. Statistical Software for Environmental Applications for Data Sets with and without Nondetect Observations. Software and supporting documentation. This version is no longer available online but has been replaced with ProUCL 5.1 (with same functionality). https://www.epa.gov/land-research (accessed 3/9/2020).
94. EU (European Union) 2011. Commission Regulation (EU) No 1259/2011 of 2 December 2011 amending Regulation (EC) No 1881/2006 as regards maximum levels for dioxins, dioxin-like PCBs and non dioxin-like PCBs in foodstuffs. http://data.europa.eu/eli/reg/2011/1259/oj
95. Health Canada 2012. Federal Contaminated Site Risk Assessment in Canada, Part I: Guidance on Human Health, Preliminary Quantitative Risk Assessment (PQRA), Version 2.0. First published 2010, revised 2012. Government of Canada 52 p. https://www.canada.ca/en/health-canada/services/food-nutrition/food-safety/chemical-contaminants/maximum-levels-chemical-contaminants-foods.html (accessed 13/9/2020)
96. OMOECC (Ontario Ministry of Environment and Climate Change) 2015. Guide to Eating Ontario Fish, 2015–2016. Toronto, ON. https://dr6j45jk9xcmk.cloudfront.net/documents/4460/fishguide2015-final-aoda-en-final.pdf (accessed 12/2/2018).
